# Poster Session II - A288 TUBULOINTERSTITIAL NEPHRITIS: A RARE EXTRA-INTESTINAL MANIFESTATION OF INFLAMMATORY BOWEL DISEASE

**DOI:** 10.1093/jcag/gwaf042.288

**Published:** 2026-02-13

**Authors:** A Cohen, A Griffiths

**Affiliations:** The Hospital for Sick Children Division of Gastroenterology Hepatology and Nuturition, Toronto, ON, Canada; The Hospital for Sick Children Division of Gastroenterology Hepatology and Nuturition, Toronto, ON, Canada

## Abstract

**Background:**

Children with inflammatory bowel disease (IBD) are at increased risk of developing kidney disorders, with one such example being tubulointerstitial nephritis (TIN). While many etiologies have been explored, TIN in the context of IBD is often attributed to the patient’s medications (e.g., 5-aminosalicylates or biologics), and these medications may therefore be discontinued.

**Aims:**

In this case report, we describe a teenager who presented with end-stage renal disease secondary to TIN and was found to have unrecognized, untreated Crohn’s Disease (CD).

**Methods:**

Data relevant to this case was extracted from our electronic medical record with the patient’s consent.

**Results:**

A previously healthy 16-year-old male presented to the hospital with a one-year history of fatigue and weight loss, and a one-week history of non-bloody diarrhea and vomiting. He was found to have previously undiagnosed chronic renal failure, and kidney biopsy confirmed a diagnosis of TIN with severe interstitial fibrosis and tubular atrophy. Infectious work-up for his diarrhea was negative and fecal calprotectin was elevated. Upper and lower endoscopy revealed patchy colonic inflammation with diffusely scattered ulcers, as well as microscopic evidence of several granulomas, lymphocytic esophagitis, focal enhanced gastritis, and chronic active duodenitis. He was diagnosed with CD and treatment was initiated with infliximab, after which he achieved clinical remission and mucosal healing. Despite deep remission of his CD, dialysis-dependent renal disease persisted, leading to kidney transplant one year later. Infliximab was discontinued pre-transplant; post-transplant immunosuppression included tacrolimus, mycophenolate mofetil, and low-dose prednisone. Two years later, the patient remains in clinical and biochemical remission, with normal fecal calprotectin and endoscopy showing no signs of chronic or active inflammation.

**Conclusions:**

While TIN has been previously described in patients with IBD, it has been difficult to determine whether this is an extra-intestinal manifestation or a consequence of IBD therapy. While TIN has been convincingly linked to 5-aminosalicylates, more recent reports have sought to draw a connection between TIN and biologics such as infliximab and vedolizumab. Making such connections erroneously may lead to unnecessary drug withdrawal and ensuing uncontrolled gastrointestinal inflammation. Our case emphasizes the fact that TIN may occur as a rare comorbidity of IBD in the absence of causative medications. Further research is needed to clarify the underlying mechanisms linking these two diseases.

**Funding Agencies:**

None

